# Comment on Oldenburg, M., Jensen, H.J. Stress and Strain among Seafarers Related to the Occupational Groups. *Int. J. Environ. Res. Public Health* 2019, doi:10.3390/ijerph16071153

**DOI:** 10.3390/ijerph17041141

**Published:** 2020-02-11

**Authors:** David Lucas, Olaf Chresten Jensen, Brice Loddé, Richard Pougnet, Jean-Dominique Dewitte, Dominique Jegaden

**Affiliations:** 1 ORPHY Laboratory, University Brest, F-29200 Brest, France; brice.lodde@chu-brest.fr; 2 Occupational and Environmental Diseases Center, Teaching Hospital, F-29200 Brest, France; richard.pougnet@chu-brest.fr (R.P.); jean-dominique.dewitte@chu-brest.fr (J.-D.D.); 3 French Society of Maritime Medicine Brest, F-29200 Brest, France; dominique.jegaden@wanadoo.fr; 4 Centre of Maritime Health and Society, University of Southern Denmark, 6700 Esbjerg, Denmark; ocj@health.sdu.dk; 5 Laboratoire d’Etude et de Recherche en Sociologie (EA 3149), Université de Brest—Bretagne Occidentale, F-29200 Brest, France

Recently, Oldenburg M published an article in the journal “Communication” titled “Stress and Strain among Seafarers Related to the Occupational Groups” [1]. They held a study on 323 seafarers on 22 container ships and used a questionnaire built from a previous study on stressors of seafarer [2]. To our knowledge, there is a lack of a methodology and discussion in this study.

In this questionnaire, they included specific job-related factors such as the shipping route, job duration at sea and also physical stressors (noise, seasickness) or psychosocial stressors (shifts, social problems due to migration). Questions on job strain are limited to work demand and management tasks. According to this questionnaire, nautical officers were more frequently mentally stressed, and the authors linked this to higher maximum working hours and higher work demand. However, the questionnaire used was not evaluated and compared to validated questionnaire such as the job demand–control–support model of Karazek [3]. Possibly due to the very specific questions, no comparison with other working populations (port workers, inland engineers, etc.) was done, which decreases the relevance of the data and results of this study. 

Interestingly, they found a lack of sleep in respondents, with a daily effective sleep duration of 5.0 h. In this paragraph, the authors talked about chronic fatigue and its relationship with human error in shipwrecks; however, they did not discuss boredom. Boredom is generally considered an emotion; according to Hill and Perkins (1998), boredom occurs when we are faced with a monotonous life combined with frustration [4]. This combination is often found among seafarers, because of the monotony of work on board—routine deck-work or using machinery, being on watch, or doing maintenance tasks—especially at sea. This boredom at work is a source of stress and addiction, according to data from the literature [5,6,7,8,9]. Most of the studies on boredom in working populations have been performed for air and road transport [9,10].

In a recent study, Jegaden et al. interviewed 80 seafarers (40 officers and 40 crew members) and 63 office staff face-to-face with three validated questionnaires: the Boredom Proneness Scale (BPS), the Hospital Anxiety Depression Scale (HADS), and the Job Content Questionnaire (JCQ) [11]. Between the two groups of seafarers, they found significant differences in the boredom disposition score and external stimulation score (respectively, Fischer test values of 5.02 and 8.19; p test values of 0.02 and 0.05). The results of the JCQ by Karasek are significantly different regarding the averages of the job demand and the job control, whereas there is no difference in social support [3]. Compared to the officers and the office staff, the crew have significantly lower job demand and job control results, which ranks them in the “passive” category (51%). On the other hand, the percentage of “actives” is significantly higher among officers (30% vs. 5.1%).

For seafarers, especially in container ships, working tasks and stress levels depend on the voyage episode. Oldenburg et al. described a higher stress level during port stays and less at sea.

According to these different studies, we can divide stress and strain among seafarers with higher job demands and management tasks during port stays and arrival–departure. At sea, the job demand was less, but monotony and repetitive tasks bring about a risk of boredom. Many works have demonstrated a strong link between boredom proneness and depression [12,13,14].

In his review of the literature into the mental health of seafarers, Iversen found that, from 1960 to 2009, 5.9% of total deaths were as a result of suicide and 13.1% of deaths were due to illness [15]. 

To prevent mental health disorders in seafarers, all characteristics of organization at sea, at port and in different types of vessels have to be included in prevention programs [16], and more research into boredom and stress factors in seafarers and fishermen have to be developed. 
